# Development of Social Skills in Kindergarten: A Latent Class Growth Modeling Approach

**DOI:** 10.3390/children8100870

**Published:** 2021-09-29

**Authors:** Yan-Tong Zhu, Xiang Li, Dan-Dan Jiao, Emiko Tanaka, Etsuko Tomisaki, Taeko Watanabe, Yuko Sawada, Zhu Zhu, Ammara Ajmal, Munenori Matsumoto, Tokie Anme

**Affiliations:** 1 School of Comprehensive Human Science, University of Tsukuba, Tsukuba 3058577, Japan; zyt199431@gmail.com (Y.-T.Z.); lixiangdufl@gmail.com (X.L.); jdd2013112@gmail.com (D.-D.J.); zhuzhu881231@yahoo.co.jp (Z.Z.); ammara.ajmal6@gmail.com (A.A.); m_matsumoto@g.t-junshin.ac.jp (M.M.); 2 Faculty of Nursing, Musashino University, Tokyo 2028585, Japan; warakott@gmail.com; 3 Faculty of Nursing, Keio University, Tokyo 1088345, Japan; ettsukot@gmail.com; 4 Faculty of Nursing, Shukutoku University, Chiba 2608701, Japan; taeko.watanabe@soc.shukutoku.ac.jp; 5 Faculty of Health Medicine, Morinomiya University of Medical Sciences, Osaka 5598611, Japan; ysawa1110@yahoo.co.jp; 6 Faculty of Medicine, University of Tsukuba, Tsukuba 3058577, Japan

**Keywords:** social skills, home rearing environment, kindergarten children, latent class growth analysis

## Abstract

Social skills acquired during early childhood are often the foundation for success later in life. Using a nationwide survey dataset in Japan, this study aims to explore the multiple growth trajectories of social skills among children in kindergarten by using a latent class growth modeling approach. It also examines whether, and to what extent, the home-rearing environment at early age predict trajectories of social skills development. Children in this study were assessed on social skills at three waves, four home-rearing environment dimensions (human stimulation, social stimulation, avoidance of punishment, and social support for parenting) and demographic background were measured at wave 1. The results indicated that three distinct growth trajectories of social skills existed during kindergarten: high increase levels, moderate increase levels, and decreased levels. The avoidance of punishment and children’s gender significantly predicted the growth trajectories of social skills. Thus, the results suggest that more attention should be paid to the home-rearing environment and boys.

## 1. Introduction

Social skills are characterized as learned, socially acceptable behaviors that allow an individual to effectively communicate with others, while avoiding socially unacceptable responses (Gresham and Elliott [1]). The development of social skills is recognized as a fundamental aspect of building successful relationships with others and has shown a positive link to children’s academic achievement, intellectual and behavioral development, and school adaptation (Wentzel et al. [2]; Hukkelberg et al. [3]; McIntyre et al. [4]). Conversely, low levels of social skills at early stages are associated with maladjustment problems, low self-esteem, and poorer mental and physical health (Arnold et al. [5]; Ali. [6]; Ke et al. [7]). Many experts believe that the preschool years are a crucial time for children to improve their social skills, since most children begin to learn how to manage themselves in order to communicate effectively with peers and teachers (Fabes et al. [8]). Therefore, it is important to clarify the development of social skills during the kindergarten period, identify children manifesting social skill deficits, provide interventions aimed at enhancing their social skills, and be diligent in taking appropriate preventative steps.

Many previous studies have outlined the characteristics of children’s social skills development (Lamont and Horn, [9]; Sørlie et al. [10]; Hajovsky et al. [11]; DiDonato, [12]). Sørlie et al. [10] suggested that children from 4th grade to 7th grade have three distinct trajectories, one with steady average scores through time, and the other two with high initial and dropping scores, as well as low initial and increasing scores. Lamont and colleagues (2013) also identify the three distinct social skills growth trajectories from kindergarten to third grade: stable class, increase class and decreasing class. DiDonato [12] discovered children from kindergarten to 5th grade have two unique trajectories of social skill: a higher-level trajectory with a marginally significant curved shape and a consistent moderate-level trajectory. Hajovsky et al. [11] found that from kindergarten to sixth grade, boys’ social skills showed a linear decline over time, whereas girls’ social skills did not change significantly over time. However, very few studies have focused on the trajectory of social skills development during the kindergarten period, which is considered a crucial time for children to improve their social skills (Kramer et al. [13]). During this time, teachers and peers expect children to begin displaying social skills while dealing with increased environmental demands (Stright et al. [14]). According to socialization theory, preschool-aged children learn how to be good as they become older, increasing the frequency of prosocial behaviors (Eisenberg et al. [15]). Simultaneously, evidence suggests that children’s social skills differentiate and show heterogeneity (Flynn et al. [16]). Within this context, the patterns of social skills development during the kindergarten (typical age of 3–6 years) period need to be further explored.

Previous studies have shown that home-rearing environment are the growth-promoting factors of social skills development (Anme and Segal [17]). The home-rearing environment can be defined as the degree of organization in children’s surroundings in relation to human and physical resources, particularly their parents, and the quality of close relationships in their environment (Anme et al. [18]). The development of social skills initially starts at home at the interpersonal level through interactions with parents (Olcer and Aytar [19]). Research has demonstrated that high quality of home rearing environment can contribute to social skills development during early childhood (Takahashi et al. [20]). Anme and Segal found a link between parenting quality and children’s social development, particularly social skill, and communication abilities [17]. Children learn social skills via parents’ interactions, parental modeling and practices, parent-children attachment and warm relations, and the experiences and opportunity to develop various social skills that parents provide and organize (Grusec and Davidov [21]; Parke et al. [22]; Reich and Vandell [23]; Denham et al. [24]). The increase in parents’ involvement over time was related to concomitant improvement in children’s social skills and decline in problem behaviors (Nokali et al. [25]). Previous studies also found that discipline and punishment imposed by parents significantly predicted children’s future social skills based on Bandura’s social learning theory (1973), When parents spank their children, they model aggressive behavior, which their youngsters replicate in disagreements with friends and siblings (Altschul et al. [26]; Tompkins and Villaruel [27]). Within this context, we hypothesis that higher quality of home-rearing environment may affect growth patterns of social skill during kindergarten.

Many studies have shown that children’s social skills development was important and has individual differences during the kindergarten period (Flynn et al. [16]; Greene [28]; Takahashi et al. [20]). Traditionally, longitudinal studies have used variable-centered methods to examine the growth curve of children’s social skills development and its relationship with its predictors. However, few studies have focused on the trajectory of social skills development during kindergarten. As a person-oriented approach, the latent class growth model is an analytical method to summarize data across multiple time periods and to characterize heterogeneous patterns within distinct groups (Jung and Wickrama, [29]). Utilizing LCGA can clarify the distinct growth trajectories of children social skill development during kindergarten and help us explore the related factors. Therefore, this study used latent class growth analysis (LCGA) to identify distinct growth trajectories of social skill during kindergarten; And further investigated how the home-rearing environment and demographic characteristic affect growth patterns.

## 2. Materials and Methods

### 2.1. Participants

This study was part of a nationwide cohort study called the ‘Child Care Cohort Study’ (CCC). Beginning in 1998, the CCC study sought to investigate the factors associated with child development and quality of life. The goal of this cohort study is to see if there was a link between the quality and quantity of center-based care and children’s social competence and vocabulary/motor/intelligence development over time, and the childrearing environment provided by parents and children’s development over time. All government authorized child day-care and night-care centers across Japan participated in it. Follow-up studies were conducted every year to investigate the factors associated with these aspects. Japanese preschools are typically three-year program for children age 3–6. As the present study was longitudinal, data from 2017 and 2019 were used. In the 2017 sample, 642 Japanese children in their first kindergarten year (typical age of 3–4) were recruited, the baseline return rate was 71.5%, giving data from 26 child-care facilities. Children with missing data on all measurement occasions was excluded in this study. Overall, all 459 children responded in the baseline were included in the analyses. The mean age of the baseline year children is 47.05 months.

### 2.2. Procedure

In the winter of each year, data was collected with signed informed consent. Receiving consent from children’s parents, we recruited children at the beginning of the project. The home-rearing environment of children and demographic characteristics was evaluated through a self-administered survey for parents at 2017. Social skills were assessed at three time points between 2017 and 2019 in the child-care facilities by trained staffs.

### 2.3. Measures

#### 2.3.1. Social Skills

Teachers’ reports of children’s social skills development were assessed from 2017 to 2019 using the Social Skill Scale (SSS) (Anme et al. [30]), which showed good reliability and validity (Anme et al. [30]; Hosokawa and Katsura [31]; Takahashi et al. [32]). The reliabilities of this study were 0.942, 0.957, 0.950 in three time points, respectively. It measures social skills through 30 items regarding “cooperation,” “self-control,” and “assertion,” all of which influence later social adaptation (Gresham and Elliott [33]). Each item was rated on a 3-point scale ranging from 0 (not at all) to 2 (often), indicating how frequently the caregivers thought children in their classroom exhibited each social skill and/or problem behavior. A higher score indicated a higher level of social skills.

#### 2.3.2. Home-Rearing Environment

The Index of Child Care Environment (ICCE) was used to measure the home-rearing environment (HRE) (Anme et al. [18]), that included 13 items in four dimensions: five questions regarding human stimulation (e.g., “How often do you play with your children?”), three regarding social stimulation (e.g., “How often do you go shopping with your children?”), two regarding avoidance of punishment (e.g., “How many times did you spank your child last week?”), and three regarding social support (e.g., “How many times do you have a chance to talk with your spouse/partner about your child?”). The ICCE is an established and valid screening instrument, given the positive correlations observed between it and child development in previous studies (Anme et al. 18]). The correlation coefficients for between the total score and each subscale of the ICCE and the HOME “total score,” “human stimulation,” “social stimulation,” “avoidance of punishment,” and “social support,” were 0.76, 0.78, 0.82, 0.82, and 1.00, respectively, and showed the high reliability (α = 0.891) (Anme et al. [18]; Bradley and Cladwell [34]). For two of the items, “support for childcare” and “have a consultation,” the response ranges were measured in a binary manner (1 = no, 2 = yes); the other 11 items were measured on a five-point scale (1 = rarely, 2 = 1–3/month, 3 = 1–2/week, 4 = 3–4/week, 5 = almost every day). To create scores that indicate whether home rearing environment is of higher or lower quality in each subscale, in the data analysis, parents who answered “1 = rarely,” were regarded as an unfavorable group and coded as 0, while those who answered “2 = 1–3/month, 3 = 1–2/week, 4 = 3–4/week, or 5 = almost every day” were regarded as a favorable group and coded as 1. Two items regarding avoidance of punishment were reversed-coded: parents who answered that they did not spank children in the past week or when they made a mistake, were coded 1 (Chen et al. [35]).

#### 2.3.3. Covariates

Based on previous studies, gender, family structure, and siblings were considered as covariates (Anderson-Butcher et al. [36]; Huang et al. [37]; Sang Nelson [38]. All covariates were considered as categorical variables: gender (boys = 0, girls = 1), family structure (nuclear family = 0, extended family = 1), and siblings (no siblings = 0, having siblings = 1).

### 2.4. Ethical Considerations

This study was approved by the University of Tsukuba (1657). All participants were informed about the study’s objectives and process and made aware that they had the right to withdraw from the study at any time. All participants gave their written informed consent to participate in the study and data were kept confidential and private and all participants’ identities were kept anonymous.

### 2.5. Statistical Analysis

The characteristics of children in their first year of kindergarten were confirmed using descriptive statistics. Latent growth curve modeling (LGCM) and Latent class growth modeling (LCGM) was performed utilizing Mplus 8.6 (Muthén and Muthén, Los Angeles, CA, USA). This study applied LCGM model, which is a special type of Growth mixture model (GMM), that assumes all individual growth trajectories in one class are homogeneous and allows only across class (Nagin [39]). Compared with GMM, LCGM can result in a more parsimonious model, and more helpful with a small sample size (Jung and Wickrama [29]; Zhang et al. [40]). We conducted our model in the following steps: First, LGCM was performed to examine the general trajectory of social skills development (e.g., linear growth or nonlinear growth) using data from three waves. Full-information maximum likelihood (FIML) and robust standard errors (MLR in Mplus) was used to deal with missing data. Because of only three waves of social skill data, we conducted three model to explore the overall social skill trajectories: no-growth mode, linear growth model and nonlinear growth model (e.g., latent basis model). Model fitness was assessed by calculating and comparing chi-square values, comparative fit index (CFI), Tucker–Lewis index (TLI), and root mean square error of approximation (RMSEA). Second, unconditional LCGA was used to categorize distinct classes based on the social skills trajectories. We estimated trajectories of social skill without predictors in the model and determined the number of classes according to the Akaike information criterion (AIC), Bayesian information criterion (BIC), sample-adjusted Bayesian information criterion (aBIC), entropy, Lo-Mendell-Rubin likelihood ratio (LMR), and bootstrapped likelihood ratio tests (BLRT), as well as theoretical justification and interpretability. Then, optimal class membership was saved and then merged with the original data. Finally, we added covariates in the conditional LCGM and explored their relationship with the trajectories, a multinomial logistic regression analysis was performed to identify predictors of classes. Our analyses and reporting of results were guided by the Guidelines for Reporting on Latent Trajectory Studies (van de Schoot et al. [41]).

## 3. Results

### 3.1. Descriptive Statistics of the Study Sample

Descriptive statistics for the background characteristics of children, home-rearing environment, and child social skill in kindergarten are presented in Table 1. Of the total sample, 52.7% (*n* = 242) were boys and 47.3% (*n* = 217) were girls. While a total of 271 (59.0%) children lived in nuclear families, 188 lived in extended ones (41.0%). Out of the children, 64.7% (*n* = 297) had siblings, while 35.3% (*n* = 162) did not. The average scores of the four aspects of the home-rearing environment in 2017 were 4.75 ± 0.57 (human stimulation), 2.57 ± 0.59 (social stimulation), 0.92 ± 0.67 (avoid of punishment), and 2.67 ± 0.59 (social support). The mean social skills scores were 42.98 ± 10.34 in 2017, 48.14 ± 11.12 in 2018, and 51.60 ± 9.46 in 2019. Bivariate correlations among the main variables are shown in Table 2.

### 3.2. Overall Trajectory of Social Skill

No-growth, linear growth, and nonlinear growth model were conducted to determine the overall trajectory patterns. The no-growth model showed the poor model fit: χ^2^ (4) = 103.48, *p* 0.001, CFI =0, TLI = 0, and RMSEA = 0.233, SRMR= 0.339. The linear growth model provided good fit to the data: χ^2^ (1) = 1.48, *p* = 0.22, CFI =0.988, TLI = 0.965, and RMSEA = 0.032, SRMR= 0.019. The linear growth model was significantly better than the no-growth model in the Satorra-Bentler chi-square test, χ^2^ (3) = 90.78, *p* 0.001, and no worse than latent basis growth model, χ^2^ (1) = 1.48, *p* = 0.22. Therefore, we selected the linear model in the next analysis. The intercept of 42.943 (*p* 0.001) and average slope of 8.930 (*p* 0.001) showed that children social skill varied in the initial levels and the rate of changes.

### 3.3. Heterogeneity in Social Skill Trajectories

The fit information for the unconditional latent class growth model is presented in Table 3. The 2-class, 4-class, 5-class, and 6-class models did not replicate the best LMR value, and LMR and BLRT in 3-class model were both significant (*p* 0.05, *p* 0.001), suggesting that 3-class model provided better model fit than 2-class model and 4-class, 5-class, and 6-class models. LMR shows the model fit between k and k−1 model, the significant LMR *p* value imply that current model has a better model fit than k−1 class model. Hence, the 3-class model were selected as the best fit model for the observed data. The proportion of individuals within each class was 74.7% (*n* = 343) in class 1, 5.3% (*n* = 24) in class 2, and 20.0% (*n* = 92) in class 3. 

**Figure 1:**
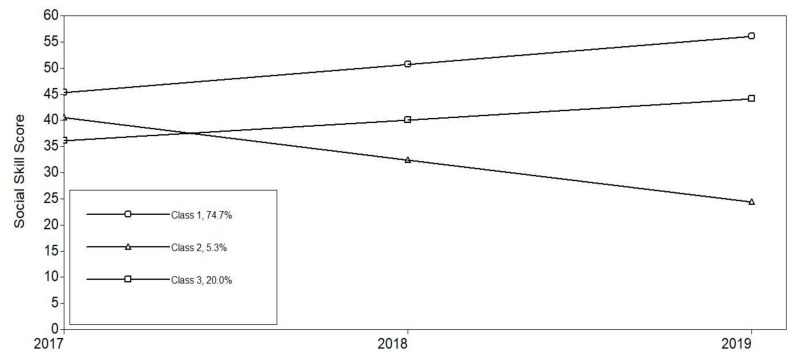
Class trajectories of social skills development.

As shown in Figure 1, Class 1 was termed “high-increase class,” that had an average initial social skills score of 45.30 (*p* 0.05) and showed a relatively modest increase compared to the Class 2 (slope = 5.37, *p* 0.05). Class 2 was named “decrease class,” as members began with an average social skills score of 40.48, that was significantly different from zero (*p* 0.05), and showed total decrease across the entire time period (slope = −8.04, *p* 0.05). Class 3 was named “moderate-increase class,” that had an average initial socials skill score of 36.07 (*p* 0.05) and subsequently increased (slope = 4.01, *p* 0.05).

### 3.4. Predictors of Identified Trajectories

Table 4 shows the impacts of the predictors for social skill trajectories using multinomial logistic regression. The finding indicated that gender was statistically significant in predicting the trajectories of latent class membership. Girls tended to belong to high-increase class than moderate-increase class as compared to boys (β = 0.845, OR = 2.328, *p* 0.01). Children whose parents never used spanking and educated children in other ways when children make mistake were more likely to belong to high-increase class than moderate-increase class as compared to those who often did (β = 0.884, OR = 2.421, *p* 0.001). However, no results were found between high-increase class and decrease class, as well as moderate-increase class and decrease class.

## 4. Discussion

This study examined children’s social skills development during the kindergarten period and identified the predictors of this growth trajectory using a latent class growth approach. On average, children social skill in kindergarten increased linearly over time, which is a similar finding to a recent study of multicultural adolescents. In current study, the trajectory of social skill in kindergarten evaluated by latent growth curve model started at 42.943 points in the first year of kindergarten and increased by 8.930 points every year in kindergarten. Given the heterogeneity of longitudinal trajectories of children social skill over time, we found that a 3-class model best described children’s social skills development during this period. The three groups outlined were consistent with a previous study (Takahashi et al. [20]). We also found that three distinct social skills development trajectories high-increase class, moderate-increase class, and decrease class during the kindergarten period. Few studies clarified children social development in kindergarten. The three identified trajectories in this study can help parents, childcare staff and professionals have a better understand the social skill development and pay more attention to children whose social skills are decreasing. 

In this study, demographic characteristics and home-rearing environment were explored to predict social skills growth trajectories. Girls were more likely to belong to the “high-increase class,” which is consistent with previous studies in the teacher-rated scale (Chan et al. [42]; Hajovsky et al. [11]). Studies have shown that girls are more likely to possess higher social skills and academic competence, boys have often more problem behaviors, which can approve the results in this study (Gresham and Elliot [33]; Abdi [43]; Mohamed [44]). Gender difference in social skill trajectories can be explained from a social perspective according to social learning theory (Bandura [45]). Once children are identified as belonging to a gender group, reinforcements from adults and peers are applied in different ways when children’s behaviors conform to gender-based expectations (Hajovsky et al. [11]). Teachers may have gendered expectations in the classroom, with girls and boys receiving differing feedback on appropriate classroom behavior (Koch, [46]), children showed different cross-gender toy choice and friends’ selection (Carter and Levy [47]). A study also clarified positive and healthy teacher–child relationship and teacher-children interactions in the classroom is more advantageous for females than males (Mohamed [44]), and may naturally enhance children’s classroom engagement and elicit more prosocial children behaviors (Hajovsky et al. [11]). Our study extended the literature with exploring gender difference in the longitudinal social development, and imply girl tend to be in the higher lever social skill class during kindergarten. This finding is essential, as it gave the evidence to the theory and gender difference in distinct social skill growth trajectories. And it also implied more supports, prevention and intervention methods should be provided for boys in kindergarten.

This study indicates that children whose parents avoid punishment tend to be in the “high-increase class.” Tompkins and Villaruel [27] found that parental punishment is significantly related to children’s social competence through parent–child exchange. This can be explained by several theoretic framework. The moral development theory established by Hoffman [48] asserts that although strong punishment can lead to compliance in young children, it can also produce fear, and obedience is often temporary because children are more concerned with their personal fear and anxiety than with internalizing the message that the behavior is unacceptable. Hence, punishment interferes with children’s moral internalization of right behavior and does not foster prosocial behavior because it promotes children to focus on self-concern (Hoffman [49]). In relation to the social learning theory outlined in the introduction, punishment would be adversely or unrelated to social skills because punishment does not provide knowledge about socially competent behavior or model the behaviors we want to see children engage in (Tompkins and Villaruel [27]). According to social control theory, parents’ use of harsh punishment, such as corporal punishment, is supposed to inhibit moral internalization by weakening the parent-child attachment link (Gottfredson and Hirschi [50]). Children who lack an attachment bond with their parents will struggle to identify with them and internalize their values as well as those of society, resulting in low self-control and an unwillingness to consider long-term consequences, leading to aggressive, antisocial, delinquent, or criminal behavior. Because they provide for quick and easy fulfillment of desires (Gottfredson and Hirschi, [51,52]; Sampson and Laub [53]; Gershoff [54]). Thus, parental punishment erodes parent-child relationships and reduces a child’s motivation to internalize his or her parents’ and society’s ideals, thereby resulting in poor self-control; this process, in turn, has a negative impact on children’s social development (White and Status [55]). This study is the first study that showed the longitudinal effect of parent’s punishment on children’s social skill growth trajectories in kindergarten, less punishment can contribute to high social skill development in kindergarten. This finding can help parent avoid to use punishment and use others parenting strategies when children make mistakes.

This study sheds new light on the growth patterns and trajectories of social skills during kindergarten, that provides new and unique perspectives for our understanding of the development of social skills with regard to factors such as demographic characteristics and home rearing environment. In addition, it highlights the utility of LCGA in elucidating the heterogeneity in social skills development among Japanese preschoolers; to our knowledge, our study is the first to examine the association between social factors and their predictors among a sample of Japanese kindergarten children using LCGA. The most significant strength of this study is that we provide an understanding for modifying the home-rearing environment, that can enhance the development of social skills during the kindergarten period.

Nevertheless, this study had several limitations. First, this study utilized teachers’ reports social skill care, while some studies examine children social skill through parent-rated scale. Both measurements are a single source of information that may not capture children’s social skills in other environments. In future studies, we suggest combining these methods for comprehensive measurements. Second, due to the limited sample size and missing rate, the power of the results might be reduced. We suggest that future studies use a larger sample size to identify the growth trajectories of social skills development. Third, although the current study included a number of significant demographic variables related to social skills, other factors, such as the parents’ education level, marital status, and family income, may also have a crucial effect on children’s social skills development (Huang et al. [37]). Researchers may be able to learn more about how children’s social skills develop in their rating settings by taking these factors into consideration.

## 5. Conclusions

The current study identified three latent classes with different trajectories of social skills development during the kindergarten period, as well as their associations with demographic characteristics and home-rearing environment. Although the result revealed only 5.3% children in decrease class of social skill development who are more likely to be at risk for future social problems, early identification of problematic social skills development should be paid attention from parents and practitioners. The links between predictors and social skills growth trajectories highlight the importance of observing social skills growth patterns to better perceive and improve the home-rearing environment, and pay more attention to male children. Parents and professionals should focus on improving parental practices to facilitate social skills development among kindergarten children.

## Figures and Tables

**Table 1:** Descriptive statistics of the study sample.

Variables	Categories	n (%) or Mean ± SD	Observed Range	Possible Range
Background characteristics				
Gender	Boy	242 (52.7)		
	Girl	217 (47.3)		
Family structure	Nuclear family	271 (59.0)		
	Extended family	188 (41.0)		
Siblings	Have siblings	162 (35.3)		
	No siblings	297 (64.7)		
Home-rearing environment				
Human stimulation		4.75 ± 0.57	2–5	0–5
Social stimulation		2.57 ± 0.60	0–3	0–3
Avoidance of punishment		0.92 ± 0.67	0–2	0–2
Social support		2.67 ± 0.59	0–3	0–3
Social skills score				
Social skills	2017 (*n* = 452)	42.98 ± 10.34	7–60	0–60
	2018 (*n* = 382)	48.14 ± 11.12	5–60	0–60
	2019 (*n* = 337)	51.60 ± 9.46	8–60	0–60

Note: Missing rates for human stimulation, social stimulation, Avoidance of punishment, and social support were 3.3%, 0.2%, 0.2%, and 1.7%, respectively.

**Table 2:** Bivariate Correlations among Main Study Variables.

No.	Variables	1	2	3	4	5	6	7	8	9	10
1	T1 social skill	-									
2	T2 social skill	0.302 **	-								
3	T3 social skill	0.279 **	0.388 **	-							
4	Gender	0.201 **	0.163 **	0.196 **	-						
5	Family structure	−0.026	0.038	0.015	−0.063	-					
6	Siblings	0.071	0.083	−0.014	0.030	0.120 **	-				
7	Human stimulation	0.091	0.014	0.117 *	0.056	0.008	−0.061	-			
8	Social stimulation	0.064	0.062	0.094	0.078	0.023	0.043	0.148 **	-		
9	Avoid of punishment	0.016	−0.013	0.137 *	0.052	−0.049	−0.044	0.054	0.029	-	
10	Social support	−0.002	−0.002	0.021	0.012	0.047	−0.052	0.320 **	0.103 *	0.127 **	-

Note: * *p* 0.05, ** *p* 0.01.

**Table 3:** Model fit information.

Model	AIC	BIC	aBIC	Entropy	LMR	BLRT
1	8798.61	8819.26	8803.39			
2	8637.70	8670.73	8645.34	0.854	0.068	0.000
**3**	**8567.24**	**8612.65**	**8577.74**	**0.790**	**0.034**	**0.000**
4	8524.30	8582.11	8537.68	0.815	0.108	0.000
5	8506.99	8577.19	8523.23	0.797	0.217	0.000
6	8492.29	8492.29	8511.40	0.731	0.865	0.030

Abbreviations: AIC—Akaike information criteria; BIC—Bayesian information criteria; aBIC—adjusted Bayesian information criteria; LMR—Lo-Mendell-Rubin likelihood ratio; BLRT—Bootstrapped Likelihood Ratio Tests. Note: The bold row represents the selected model.

**Table 4:** Conditional latent class growth model.

Variable	High-Increase Class vs. Decrease Class			Moderate-Increase Class vs. Decrease Class			High-Increase Class vs. Moderate-Increase Class		
	Estimated	SE	OR	Estimated	SE	OR	Estimated	SE	OR
Girls	0.261	0.549	1.298	−0.091	0.585	0.913	0.845	0.312	2.328 **
Extended family	−0.119	0.456	0.888	0.143	0.488	1.154	−0.262	0.318	0.770
Have siblings	−0.561	0.553	0.571	−1.003	0.560	0.367	0.442	0.345	1.556
Human stimulation	0.312	0.406	1.366	−0.261	0.431	0.770	0.573	0.337	1.774
Social stimulation	−0.386	0.407	0.680	−0.218	0.444	0.804	−0.168	0.274	0.845
Avoid of punishment	0.559	0.381	1.749	−0.325	0.409	0.723	0.884	0.247	2.421 ***
Social support	0.355	0.316	1.426	0.250	0.351	1.284	0.105	0.261	1.111

Note: ** *p* 0.01, *** *p* 0.001.

## Data Availability

Not Applicable.
